# Single Trial Classification of Motor Imagination Using 6 Dry EEG Electrodes

**DOI:** 10.1371/journal.pone.0000637

**Published:** 2007-07-25

**Authors:** Florin Popescu, Siamac Fazli, Yakob Badower, Benjamin Blankertz, Klaus-R. Müller

**Affiliations:** 1 Intelligent Data Analysis Laboratory, Fraunhofer Institute FIRST, Berlin, Germany; 2 Machine Learning Laboratory, Technical University Berlin, Berlin, Germany; University of Birmingham, United Kingdom

## Abstract

**Background:**

Brain computer interfaces (BCI) based on electro-encephalography (EEG) have been shown to detect mental states accurately and non-invasively, but the equipment required so far is cumbersome and the resulting signal is difficult to analyze. BCI requires accurate classification of small amplitude brain signal components in single trials from recordings which can be compromised by currents induced by muscle activity.

**Methodology/Principal Findings:**

A novel EEG cap based on dry electrodes was developed which does not need time-consuming gel application and uses far fewer electrodes than on a standard EEG cap set-up. After optimizing the placement of the 6 dry electrodes through off-line analysis of standard cap experiments, dry cap performance was tested in the context of a well established BCI cursor control paradigm in 5 healthy subjects using analysis methods which do not necessitate user training. The resulting information transfer rate was on average about 30% slower than the standard cap. The potential contribution of involuntary muscle activity artifact to the BCI control signal was found to be inconsequential, while the detected signal was consistent with brain activity originating near the motor cortex.

**Conclusions/Significance:**

Our study shows that a surprisingly simple and convenient method of brain activity imaging is possible, and that simple and robust analysis techniques exist which discriminate among mental states in single trials. Within 15 minutes the dry BCI device is set-up, calibrated and ready to use. Peak performance matched reported EEG BCI state of the art in one subject. The results promise a practical non-invasive BCI solution for severely paralyzed patients, without the bottleneck of setup effort and limited recording duration that hampers current EEG recording technique. The presented recording method itself, BCI not considered, could significantly widen the use of EEG for emerging applications requiring long-term brain activity and mental state monitoring.

## Introduction

Electro-encephalography (EEG) is the oldest brain imaging technology, and among non-invasive methods it still offers the highest temporal resolution. Far from being a mere research aide, it promises an inexpensive, risk-free means of communication and neuroprosthetic control for the severely disabled [1], [2]. Recent advances in Brain Computer Interface (BCI) research have dramatically increased the amount of information we can extract from EEG over classical averaging and neurofeedback techniques [3]. Although EEG can monitor brain events very responsively in time, it suffers from high inter-trial variability and spatial *mixing*: numerous electrical sources active at any given time in the brain are superimposed onto the scalp across distances of over 5 cm [4]. These limitations have led to the assumption that many electrodes are necessary, and that one needs to average signal features across time or repeated trials to accurately discriminate mental states.

**Figure 1:**
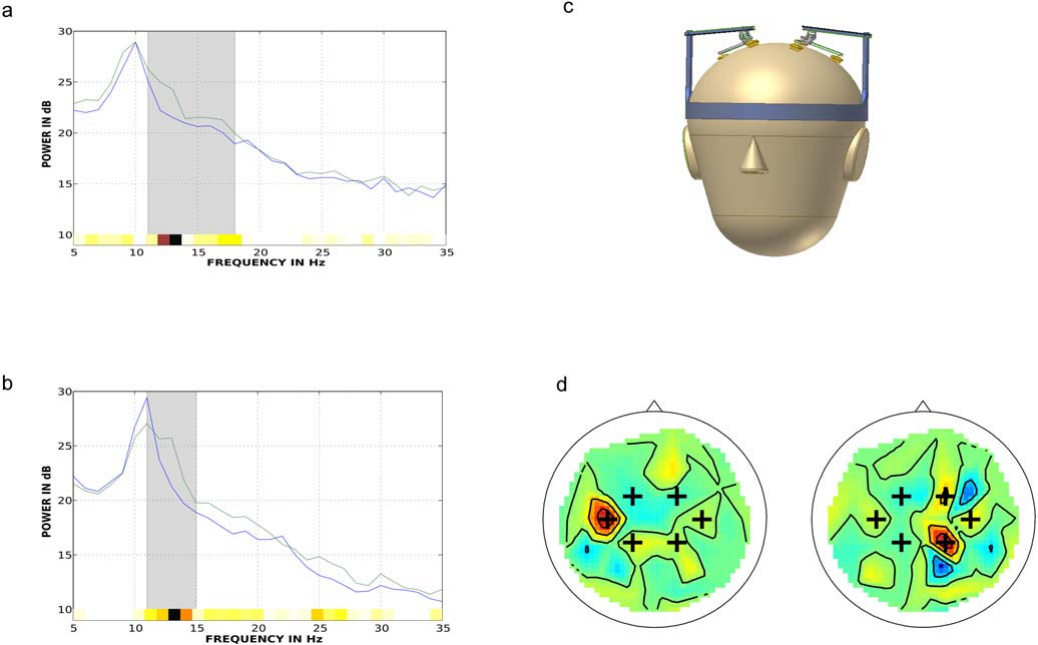
Signal spectra and electrode placement. Typical signal spectrum from proposed dry electrode (each trace corresponds to averaged spectra for each class). b) Comparable signal from conventional electrode with electrolyte gel (same subject, same conditions). c) Illustration of dry cap. d) Contralateral CSPs of left/right classes from full cap and location of 6 dry cap electrodes.

Apart from intrinsic challenges of EEG signal analysis, one of the main obstacles precluding EEG-BCI from being used in patients' daily lives is setup encumbrance. Modern EEG practice, as part of the electrode application procedure known to specialists as *montage*, requires tedious application of conductive gel between electrodes and scalp. While recordings in certain clinical applications may last up to 72 hours, they progressively degrade as the gel dries leading to a failure of about a quarter of the electrodes within 24 hours and thus requires daily maintenance [5]. We introduce a new EEG cap design with few electrodes and show that the much sought-after ‘dry electrode’ technology is surprisingly frugal and accurate enough for excellent online discrimination. Dry electrodes bypass gel application, thereby reducing set-up time. Fewer electrodes mean less time spent checking individual signal quality and adjusting the cap. Our new design (Fig. 1c) consists of only 6 dry unipolar electrodes and one dry reference electrode. The cap applies a moderate amount of pressure upon the scalp via an array of specially coated metal contacts which do not cause discomfort to the users as reported by our experimental subjects. The sparse electrode arrangement and slightly reduced ‘dry’ signal quality places the onus on robust signal processing for effective BCI.

The advent of machine learning in the field of BCI has led to significant advances in real-time EEG analysis. While early EEG-BCI efforts required neurofeedback training on the part of the user that lasted on the order of days [6] in current practice it suffices to collect data in which the patient is cued to perform one of a small set of mental tasks called *classes*. After setup and less than 30 minutes [7] of training data collection, a classification algorithm analyzes brief recordings and learns to discriminate mental tasks in less than 5 minutes of computation time, thereby relocating adaptation from the user to the computer. Robustness of BCI decoding algorithms, re-use of classifiers [8] and artifact removal have benefited from significant research effort [3].

Successful EEG analysis requires both temporal (filtering) and spatial (source-localizing) decomposition. The current Berlin Brain Computer Interface consists of a heuristic search of EEG frequency bands and time intervals which maximize class discrimination, as a temporal decomposition step. It is followed by an automatic, signal driven source localization algorithm termed Common Spatial Pattern (CSP) [9] [3] which correlates spatial activity within a class while concurrently discriminating this correlation pattern from that of another class. The final step is an algorithm which performs automatic discrimination (i.e. classification) based on *features* generated by the spatio-temporal decomposition. As has been shown [7], the frequency bands chosen, the time intervals and the spatial patterns are consistent with known neurophysiology of movement imagination, provide excellent discrimination and, as shown in this study, work well despite noise in the signal and sparse recording sites. Furthermore, the analysis method required in order to maximize information gain from EEG, as evidenced by our investigative study, can be both straightforward and effective.

## Methods

The results of our 1D cursor control paradigm [10], previously run with a full (64 gel electrode) cap [7], was repeated in this study such that dry cap performance could be compared for the same subjects. 5 healthy subjects (4 male, 1 female) participated. Two subjects were initially tested, however due to particularly thick and full hairstyle no continuously stable signal could be extracted, and thus they were excluded from the study. For 3 of the 5 selected subjects the previously collected data was used, while for the other 2 the paradigm was reproduced. All subjects were volunteers drawn from the members of the laboratory, and all had prior experience with the paradigm. As it was judged that through the use of dry electrodes there was minimal increase in physical, psychological and social risk to the subjects no further ethics board approval was needed than that already in use for gel electrodes (Charité - Universitätsmedizin Berlin Ethics Commission). As per our standard EEG procedure, which may involve skin preparation, in the unlikely case of a minor scratch, disinfectant and a first-aid kit were on hand. Subjects were instructed to end the session if they felt any discomfort. No injury of any kind occurred and no serious discomfort was reported. The subjects gave verbal consent to the eventual dissemination of results and are identified by randomized initials herein.

While EEG cap setup normally requires an attendant and about 30 minutes of preparation, the dry cap can be simply placed on the head and manually adjusted even by the subject herself in less than 2 minutes. For the ‘dry cap’ experiments a 14-channel DC amplifier set-up (BrainAmp128DC, Munich, Germany) was used (6 EEG channels and 4 bipolar artifact measure channels). In the first part of the experiment (‘calibration session’), a sequence of 80 left/right cues was presented visually by means of a letter which appears in the middle of the computer screen. The subjects were asked to imagine the cued class without moving either limbs or the eye. All subjects used left/right hand movement imagination except one subject who used left hand/ right foot imagination since the earlier study [7] predicted this combination to be optimal for that subject. The cues were presented for 3.75 seconds with an inter-cue relax interval of 1.75+/−0.5 seconds. Electro-oculo-grams (EOG) were measured using 2 standard (gel) electrodes per eye (one lateral to each eye, one above the left eye, one below the left eye) the difference between each pair being amplified as to obtain vertical and horizontal components, while surface bipolar electromyogram (EMG) electrodes where placed on the Flexor Carpi Radialis. As one of the subjects used right foot imagination for one class EMG was measured from the Gastrocnemius. Apart from off-line checks, electromyograms are monitored online and the maximal co-contraction EMG level recorded: no trials were excluded. The average dry electrode impedance measured was 78.6±30.0 KΩ.

The dry cap BCI system was thus ready for use after roughly 15 minutes: 2 for electrode preparation, 8 for calibration data collection and 5 minutes for the classifier algorithm to learn from the calibration data. For habitual use, calibration could be eliminated and classifiers reused [8]. In a second part of the experiment (‘feedback session’) subjects were asked to move a dot displayed on the screen to a target represented by a bar on either the right or left side of the screen by imagining the corresponding class. The dot movement provided continuous performance feedback to the subjects. Each subject performed 400 trials divided into 4 sets allowing him/her a brief pause for mental relaxation (See Video S1).

A semi-automatic search for the time interval of the event-related desynchronization (ERDs) and frequency band whose power discriminates most between classes for each subject generally selects the so-called *mu*- and *beta*- rhythms (8–25 Hz, Fig. 1a,b) in the motor cortex [7], [11]. The discriminating frequency band search determined a band-pass filter which attenuated signal amplitude outside these bands thereby accomplishing a temporal ‘demixing’.

The resulting filtered multivariate signals, segmented in the ERDs time interval, are used to compute two covariance matrices Σ_1_ and Σ_2_ from the calibration data. The CSP algorithm searches for a matrix *W* and a vector of *n* values 0≤*d_i_*≤1 which achieves:
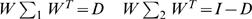
Where *n* is the number of channels and *D* is a diagonal matrix with entries *d_i_*. Using *z*-transform notation for digital signals, for any trial, the spatio-temporally de-mixed data is:
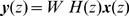
Where ***x*** is the raw EEG signal and *H(z)* is a diagonal matrix of identical band-pass filter transforms. The columns of the source to signal transform *W* ^−1^ are called the Common Spatial Patterns (CSPs). The CSP decomposition can be thought of as a coupled decomposition of 2 matrices (for 2 classes) similar to a principal components analysis yielding eigenvectors and eigenvalues. As the eigenvalues *d_i_* are equal to the power ratio of signals of class 1 by class 2 in the corresponding CSP filter (eigenvector in *i*-th column of matrix *W*), best discrimination is provided by filters with very high (i.e. near 1) or very low (i.e. near 0) eigenvalues. Accordingly CSP projections with the highest 2 and lowest 2 eigenvalues were chosen as features (*n* = 4).

The decomposed time-varying multivariate signal **y(t)** can be easily transformed into an *n*-vector of log-variances, by estimating !!!eq i 0 over a desired time window. The elements of this vector are the ‘features’ that the classifier learns to associate with a given class. The classifier used was Linear Discriminant Analysis (LDA), which assigns linear weights to features as to provide a separating hyper-plane between classes in feature space. In the ‘feedback’ sessions the time window length used was adjusted to subject preference for cursor responsiveness and ranged from 600 to 1000 msec. The speed of the cursor is proportional to the continuous linear weighted sum of features as computed by the LDA output.

In order to rule out that the reported ITRs are due to muscle artifact, we analyze whether a classifier based on EOG or EMG alone achieve a significant ITR. For this, unfiltered EOG and EMG signals were segmented into 5 windows, each 500 msec long, starting after cue presentation for feedback data. The log variance of these segments provided features (i.e. 5 segments of 2 EOG resp. EMG channels = 10 features) that were classified by LDA in a leave-one-out fashion, i.e. each segmented feedback trial is labeled by a classifier trained on all other trials.

## Results

The main object of the study was to compare the Information Transfer Rate (ITR) obtainable with the dry cap with that previously established for the full cap for an existing paradigm using the same subjects. Classification results are summarized in Table 1.

**Table 1:** Results of feedback sessions for dry vs. full cap.

Subjects	al	zg	ay	zk	aw	Average
Feedback – Gel Cap						
1D (bit/min)	24.4	13.0	22.6	8.8	5.9	14.9
correct (%)	98.0	98.0	95.0	86.8	80.5	91.7
time/trial (s)	2.1	3.9	1.9	3.0	2.9	2.8
peak (bit/min)	35.4	19.6	31.5	23.4	11.0	24.2
Feedback – Dry Cap						
1D (bit/min)	17.6	3.4	14.1	7.9	5.0	9.6
correct (%)	91.8	79.2	94.8	84.5	83.8	86.8
time/trial (s)	2.0	4.7	3.1	2.9	4.4	3.4
peak (bit/min)	36.5	14.0	25.0	23.1	16.8	23.1
Percentage difference Gel Cap – Dry Cap						
1D (%)	−27.8	−63.4	−37.6	−10.2	−15.2	−30.8
correct (%)	−6.3	−19.1	−0.2	−2.6	3.9	−4.9
time/trial (%)	4.7	−18.1	−38.7	−4.0	−34.1	−18.0
peak (%)	3.0	−28.4	−20.6	−1.3	34.5	−2.6
Feedback Classification Accuracy EEG-EOG-EMG						
EEG (%)	91.8	79.2	94.8	84.5	83.8	86.8
EMG (%)	72.3	47.5	52.2	61.1	85.8	63.8
EEG (% on EMG-)	90.4	78.7	94.3	83.5	89.9	87.4
EOG (%)	72.8	49.0	55.1	58.5	80.6	63.2
EEG (% on EOG-)	91.2	76.4	95.5	85.1	88.4	87.3
EMG (% of MVC)	2.7	1.2	1.7	1.3	0.7	1.5
EMG-fb (% of EMG-pre)	107.9	102.5	98.1	103.0	109.4	104.2

Feedback gel cap (top) reports feedback data from an earlier study (3). The first line shows the bit/min information transfer rate of 1D cursor control averaged over 8 sessions consisting of 25 trials each. The second line gives the average percentage of correct trials and the third and fourth lines provide the average time per trial and the peak performing session result. Feedback dry cap (middle) as above. Note that here 4 sessions of 100 trials each were evaluated. Also the peak performance was computed as the best 25 consecutive trials. The lower part (bottom) of the table summarizes the relative loss in performance of the respective setups for the subjects. Note that a negative sign indicates lower performance of the dry electrode cap. “% of MVC” stands for the power of feedback trials, as compared to the maximum voluntary contraction (MVC). EMG-fb stands for the EMG activity in the actual feedback trials, as compared to the preparatory phase of each feedback trial, EMG-pre.

**Figure 2:**
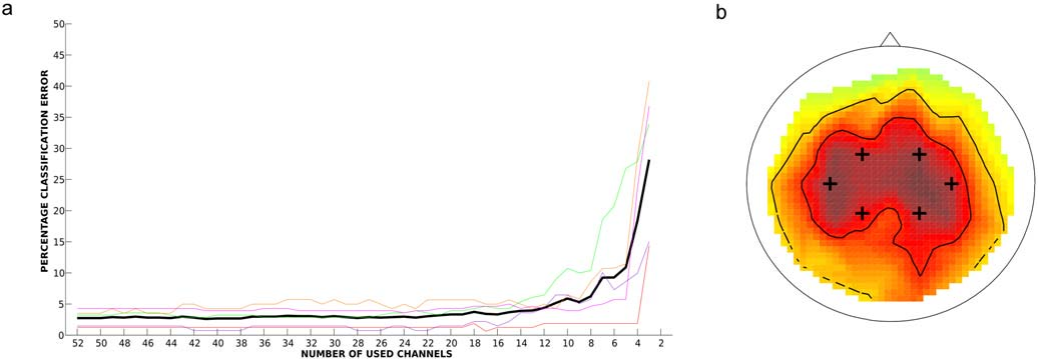
Relationship of ITR to number of electrodes and position. a) Predicted error rates vs. number of channels for different subjects (colored lines) and average (black line). b) electrode importance ranking averaged across subjects, plus dry cap electrode placement.

The locations of the 6 channels used were determined with the aid of a sensitivity analysis on full cap data similar to [12]. After a CSP matrix *W* is calculated, the row with the lowest sum of absolute values is labeled as the least-significant channel in terms of classification. After elimination of this channel from further analysis, the entire CSP/LDA classification procedure can be re-run. By performing channel elimination iteratively, we can approximate the expected error for any ‘best’ *m*<*n* channels and derive a relative ranking of channel relevance (see Figure 2).

While subject experience and proper instruction can alleviate the confounding role of EMG and EOG by encouraging performance in which no such activity can be detected (2 of the subjects had no detectable artifact) in most subjects, artifacts are unavoidable as they are involuntary in nature. The results in Table 1 (lowest part) show that classification based on EOG/EMG is either close to chance level, or much less accurate than that based on EEG. Furthermore note that in the trials in which EOG or EMG analysis erred in classification, EEG still consistently classified with the same accuracy as in other trials.

## Discussion

With only 6 dry electrodes approximately placed above the motor cortex (Fig. 1d), the information transmission rate achieved a peak of 36.5 bits/min (on par with any EEG-BCI performance reported) and is on average 30.8% slower than previous experiments with 64 wet electrode caps on the same subjects.

Despite its simplicity the CSP algorithm and extensions thereof [3], [13] remains among the highest consistent performers among the many EEG-BCI analysis techniques developed and attempted [14]. For general scientific interest, a BCI algorithm needs to do more than simply show a high ITR. Critical is the identification and description of the physiological origin of signal that provides for discrimination. It would be useful to perform ‘EEG source localization’, i.e. a spatial de-mixing of the signal which provides for electrical dipole locations back-calculated from the recorded signal. Using algorithms designed for this particular purpose, it has been shown that motor imagery based BCI does indeed localize to the motor cortex [15]. Although source localization from only 6 channels of recording cannot be done without an unacceptable loss in accuracy, we had full-cap data from the same paradigm at our disposal.

Interestingly, the CSP algorithm was originally conceived to be a signal-driven source localization technique which can locate known dipole sources [9]. As such, the primary CSP patterns of the full-cap data for left- and right- classes do indeed show highest sensitivity around the contra-lateral motor cortical areas (see Figure 1 and 2) as expected from basic motor neurophysiology. Further evidence is gained by simply asking the question: if we only had *m* electrodes available, where should they be placed in order to maximize classification? We performed a sensitivity study where the electrode that least contributed to the CSP-based classification was iteratively removed from the analysis. Results are shown in Figure 2. Note that ‘best’ electrode placement varies from subject to subject but is fundamentally fronto-parietal and bilateral (i.e. above the motor cortical areas). Note also that for at least one subject the expected 6-channel performance is low, as was confirmed in the dry cap experiment. Since potentials propagate perpendicularly from the folded cortical surface, varying anatomy and cranial electrical properties among subjects means that one cannot just place electrodes ‘above the motor cortex’ and expect maximal performance. Our study does show that such a simplifying strategy works surprisingly well, based on a ranking of electrode location relevance (see Figure 2) averaged across subjects. Individualized electrode placement will likely improve performance, but not without considerable cost, however. Further technical development of the electrode design – and specialized research - may also be necessary in order for the recording pins design to improve in such a manner that they bypass all hair-types and make consistent contact with the scalp. The subjects tested were not chosen with any such criteria in mind and good results were obtained from 5 out of the first 7 people tested.

EEG analysis, whether it is classification or localization, can be compromised by EOG and EMG even if these are produced involuntarily. Arm muscle activation or bodily movement must be considerably large in order to affect EEG [4], [16]. In our experiments, no movement is visible (See Video S1) and measured hand EMG magnitudes averaged 1.5% of maximum voluntary contraction (MVC). Note that this is not necessarily phasic activity but mostly tonic co-contraction. EMG levels during cue presentation (i.e. movement imagination) are from −1.9% to 9.5% greater than EMG levels during the brief rest period between trials. A look at the last rows of Table 1 shows that EMG classification accuracy correlates with the magnitude of this difference (on the order of 0.15% of MVC) rather than the overall EMG magnitudes. Being based on overall differences so slight, EMG affords significantly poorer classification than EEG.

EOG represents mainly ocular muscle activity but can also partially reflect facial, tongue and jaw muscle activity. As EOG electrodes are closer to the scalp than EMG electrodes, their activity, even if moderate, is more likely to represent an artifact in EEG. Note the EMG/EOG classifiers operated on feedback trial data and not calibration trial data, which may have contained other types of eye movement patterns due to the absence of visual target presentation.

Prior analysis of artifact influence in BCI experiments has shown that the type of movement can be determined earlier and more accurately in EEG than in EMG/EOG [17]. That EEG, in this study, still indicates mental states in trials and subjects in which artifact, whether EMG or EOG, cannot discriminate the mental class further reinforces the idea that the classifier responds mainly to cortical activity patterns, in a physiologically expected location and frequency range.

The implications of dry electrode technology are significant, both in terms of practicability of non-invasive BCI for the severely disabled and in terms of a robust, affordable brain imaging technique for long-term neuroscience experiments (some sessions lasted over 5 hours). Clinical applications may include daily EEG monitoring for epilepsy or narcolepsy. Regarding healthy subjects, dry-electrode BCI opens a more practical outlook for Human-Machine Interaction, for monitoring alertness, emotion or mental workload.

This study attempts to maximize the practical value of BCI from the fewest number of recording channels possible. The scientific implications of this approach are that by careful analysis and electrode placement effective functional imaging of the awake, active brain can be achieved non-invasively and in a fairly simple, cost and time-effective manner. Dry electrodes may be sparsely placed elsewhere on the scalp as to focus on other cortical areas that are not motor-related.

The state of current EEG-BCI research makes use of electrophysiological phenomena that contribute to accurate discrimination among mental states in single trials. Future research will focus on further improvements of EEG sensor and data analysis technology and strive towards simple devices that learn to adapt to a user or patient and allow communication even in highly noisy and non stationary real world scenarios.

## Supporting Information

Video S1 Sample feedback session trial set. Subject controls the cursor (a cross) to cued left/right targets (flashing bars on each side of the monitor).
(5.11 MB MOV)
